# *Lophatherum gracile* Bronghiart Suppresses Receptor Activator of Nuclear Factor Kappa-B Ligand-Stimulated Osteoclastogenesis and Prevents Ovariectomy-Induced Osteoporosis

**DOI:** 10.3390/ijms232213942

**Published:** 2022-11-11

**Authors:** Sung-Ju Lee, Seon-A Jang, Seong Cheol Kim, Jin Ah Ryuk, Hyunil Ha

**Affiliations:** 1 KM Convergence Research Division, Korea Institute of Oriental Medicine, Yuseong-daero 1672, Daejeon 34054, Republic of Korea; 2 Future Technology Research Center, KT&G Corporation, 30, Gajeong-ro, Yuseong-gu, Daejeon 34128, Republic of Korea

**Keywords:** *Lophatherum gracile* Bronghiart, osteoporosis, bone loss, ovariectomy

## Abstract

*Lophatherum gracile* Bronghiart, used in traditional herbal medicine, has many biological properties including antiviral, antipyretic, antitumor, vasorelaxation, and neutrophilic inflammatory effects. However, its modulatory effects on bone metabolism have not been investigated previously. In this study, we examined the effects of a water extract of the leaves of L. gracile (WELG) on osteoclast differentiation and bone loss, and explored its underlying mechanisms. We found that WELG inhibits osteoclastogenesis by suppressing both receptor activator of nuclear factor-κB ligand (RANKL)-induced early activation of mitogen-activated protein kinases (MAPKs) and nuclear factor-κB (NF-κB)- and RANKL-induced modulation of the positive and negative regulators of osteoclastogenesis in osteoclast precursors. In vivo study demonstrated that WELG protects against bone loss, weight gain, and fat accumulation without affecting uterine atrophy in an ovariectomy-induced postmenopausal osteoporosis mice model. In addition, photochemical analysis of WELG identified active constituents known to have bone-protective effects. Overall, the results of this study suggest that WELG can be a potential candidate for therapy and prevention of postmenopausal osteoporosis.

## 1. Introduction

Bone homeostasis is regulated by osteoclast-induced bone resorption and bone formation mediated by osteoblasts, which is a reconstituted step of bone tissue renewal for maintaining bone homeostasis in normal adult bones [1]. However, excessive osteoclast differentiation and activation can cause osteoporosis due to an imbalance between bone resorption and formation [2,3]. Osteoporosis is a metabolic bone disease characterized by extremely low bone density occurring mainly in the elderly, especially in menopausal women [2,3]. Additionally, this disease can cause bone fracture symptoms owing to enhanced bone brittleness and thinning through ultrastructural disruption [4]. Osteoclasts are giant multinucleated cells that have undergone differentiation and fusion of macrophages and monocyte-descended precursor cells and activation [5,6]. Osteoclast differentiation is initiated by receptor activator of nuclear factor-κB ligand (RANKL) and macrophage-colony-stimulating factor (M-CSF), which are engaged by various cells, including stromal cells, osteoblasts, and osteocytes [5,6]. After RANKL binds to the receptor RANK on osteoclast precursors, RANK recruits TNF-receptor-associated factors (TRAFs) [5,6]. The combined RANKL/RANK/TRAFs axis activates nuclear factor-κB (NF-κB) and mitogen-activated protein kinase (MAPK) signaling pathways, which upregulates several downstream transcription factors, including nuclear factor of activated T cells c1 (NFATc1) and cellular oncogene fos (c-Fos) [7,8,9,10,11]. Moreover, NFATc1 functions as a master transcription factor and upregulates the expression of specific osteoclastogenesis-associated genes such as tartrate-resistant acid phosphatase (TRAP), cathepsin K (Ctsk), dendritic cell-specific transmembrane proteins (DC-STAMP), and ATPase H+ transporting V0 subunit D2 (Atp6v0d2) [12,13]. Targeted regulation of these signaling pathways can prevent or treat bone loss caused by excessive osteoclast differentiation and activation [14].

*Lophatherum gracile* Bronghiart (*L. gracile*) is affiliated with the family Gramineae and is generally found in southern China. The leaves of *L. gracile* were widely used in traditional herbal medicine from old times, mostly, to treat inflammation and fever. Recently, a diversity of biological and pharmacological research on *L. gracile* has been conducted regarding topics such as its anti-viral activity, anti-cancer, fever reduction, vasorelaxation protection, and neutrophilic inflammation effects, and prevention of hyperglycemia [15,16,17,18]. In addition, several flavonoids purified from ethanolic extracts of the leaves of *L. gracile* show potent antioxidant and hepatoprotective effects [19]. However, the effects of *L. gracile* on bone metabolism have not yet been reported.

In this study, we studied the inhibitory effects of a water extract of the leaves of *L. gracile* (WELG) on osteoclastogenesis and demonstrated its fundamental mechanisms. We also examined its beneficial effects against bone loss in an ovariectomy (OVX)-induced postmenopausal osteoporosis mouse model. Our findings provide evidence for the beneficial effects of WELG on osteoclast-associated osteoporosis.

## 2. Results and Discussion

### 2.1. Suppression Effect of WELG on Osteoclast Differentiation

**Figure 1:**
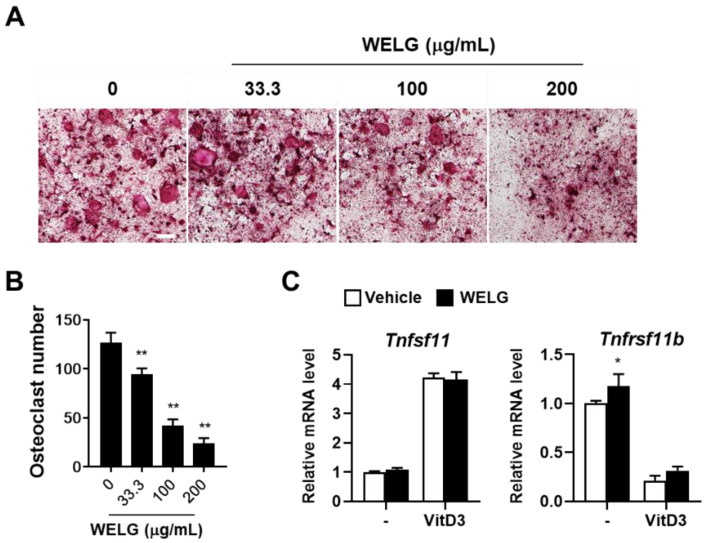
Suppression effect of WELG on osteoclast formation in co-cultures of osteocytes and osteoclast precursors. The co-cultures of MLO-Y4 and BMMs were treated with or without WELG for 5 days in the presence of VitD3 (10 nM). (**A**) Representative TRAP-staining images of the co-cultures (scale bar, 100 μm). (**B**) The number of osteoclasts, TRAP-stained multinucleated cells including more than three nuclei. (**C**) MLO-Y4 cells were treated with or without WELG (200 μg/mL) and VitD3 (10 nM) for 1 day. Tnfsf11 and Tnfrs11b mRNA expression levels were quantified by quantitative real-time polymerase chain reaction. * *p* < 0.05, ** *p* < 0.01 versus vehicle.

**Figure 2:**
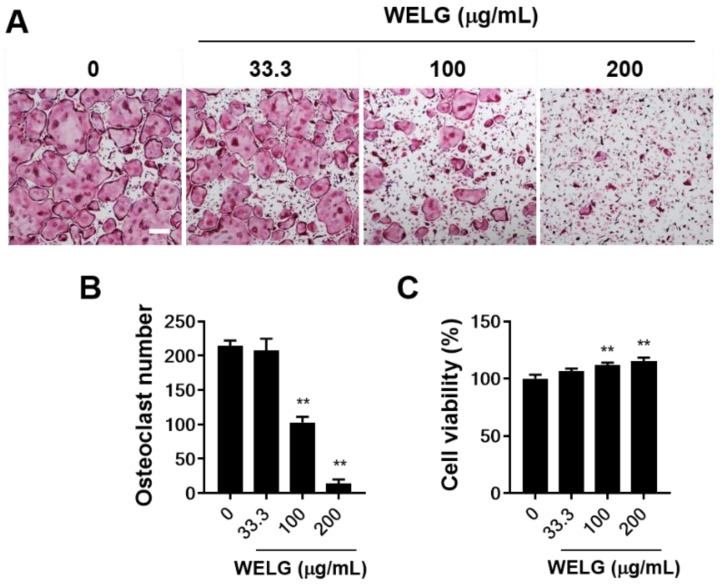
Suppression effect of WELG on RANKL-stimulated osteoclastogenesis. BMMs were treated with or without WELG in the presence of M-CSF (60 ng/mL) and RANKL (50 ng/mL) for 4 days. (**A**) TRAP-staining images of the cultures (scale bar, 100 μm). (**B**) The number of osteoclasts generated in the cultures. (**C**) Cell viability of BMMs after 24 h of treatment with WELG. ** *p* < 0.01 versus vehicle.

Osteocytes regulate osteoclast differentiation and bone resorption through the secreted essential pro-osteoclastogenic molecule, RANKL, and its inhibitor, osteoprotegerin (OPG) [20,21]. To assess the role of WELG in osteoclastogenesis, MLO-Y4, osteocyte-like cells [22], co-cultured with bone marrow derived macrophages (BMMs) were treated with various concentrations of WELG. Stimulation of the co-culture with 1α, 25-dihydroxyvitamin D3 (VitD3) increased osteoclast formation. However, treatment with WELG significantly and dose-dependently inhibited osteoclast formation in the co-culture system (Figure 1A,B). VitD3 stimulated the mRNA expression of tumor necrosis factor ligand superfamily member 11 (Tnfsf11) gene encoding RANKL and decreased the mRNA expression of tumor necrosis factor receptor superfamily member 11B (Tnfrsf11b) gene encoding OPG in MLO-Y4 cells, which was not affected by WELG (Figure 1C). Thus, we speculated that WELG might directly inhibit osteoclast differentiation independently of its action on MLO-Y4 cells to support osteoclast differentiation. To assess the direct inhibitory effect of WELG on osteoclastogenesis, osteoclast precursors, BMMs, were stimulated with RANKL to induce osteoclast differentiation. WELG suppressed RANKL-induced formation of TRAP-positive multinucleated osteoclasts in a dose-dependent manner (Figure 2A,B). Treatments of BMMs with WELG at doses showing inhibitory effects on osteoclast differentiation did not decrease but slightly increased cell viability, assessed by a Cell Counting Kit-8 (CCK-8) assay (Figure 2C). These results demonstrated that WELG directly inhibits osteoclast differentiation processes triggered by RANKL in osteoclast precursor cells.

### 2.2. Suppression Effect of WELG on RANKL Signaling Pathways during Osteoclast Differentiation

**Figure 3:**
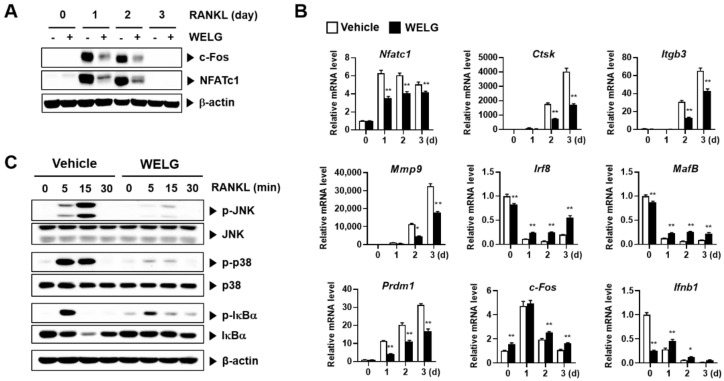
Suppression effect of WELG on RANKL-stimulated signal pathways in osteoclastogenesis. BMMs were activated by RANKL (50 ng/mL) treatment for the indicated days following pretreatment with vehicle or WELG (200 μg/mL) for 3 h. (**A**) The levels of c-Fos and NFATc1 protein on the indicated days were analyzed by Western blotting. (**B**) The levels of mRNA genes involved in osteoclastogenesis were analyzed by quantitative real-time polymerase chain reaction. * *p* < 0.05, ** *p* < 0.01 versus vehicle. (**C**) BMMs were activated by RANKL (50 ng/mL) for the indicated times following pretreatment with vehicle or WELG (200 μg/mL) for 3 h. The proteins related to RANKL-induced NF-κB and MAPK signaling pathways were analyzed by Western blotting. All tests were repeated at least three times.

RANKL-mediated signaling cascades play a key role in controlling osteoclastogenesis. RANKL binds with RANK and activates the MAPK and NF-κB pathways via TRAF adaptor proteins, which leads to upregulation of c-Fos and NFATc1 [7,8,9,10,11]. NFATc1 and c-Fos are the main transcription factors for osteoclast formation as well as differentiation and upregulation of osteoclast-specific genes, including TRAP, matrix metallopeptidase 9 (Mmp9), integrin beta 3 (Itgb3), and Ctsk [13,23]. To explore the inhibitory mechanism of WELG on osteoclast differentiation, we examined the effects of WELG on RANKL-induced expression and activation of these molecules. Treatment of BMMs with RANKL increased NFATc1 mRNA and protein expression, which was significantly inhibited by WELG (Figure 3A,B). Subsequently, WELG suppressed RANKL-induced upregulation of osteoclastic marker genes Ctsk, Itgb3, and Mmp9 (Figure 3B). Interferon regulatory factor 8 (IRF8) and v-maf avian musculoaponeurotic fibrosarcoma oncogene homolog B (MafB), known as the negative osteoclast transcription factors, suppress the transcriptional activity of NFATc1 [24,25]. These negative osteoclast transcription factors are negatively controlled by transcriptional repressors such as positive regulatory domain zinc finger protein 1 (Prdm1; encoding Blimp1) [26]. WELG significantly suppressed a RANKL-induced decrease in Irf8 and MarB expression and increase in Prdm1 expression (Figure 3B). RANKL-induced protein, but not mRNA, expression of c-Fos, an upstream transcription factor of NFATc1 for osteoclastogenesis [23], was also inhibited by WELG (Figure 3A,B). Because interferon-β (IFN-β) has been shown to inhibit RANKL-induced protein, but not mRNA, expression of c-Fos [27], we investigated the effect of WELG on Ifnb1 expression. Although WELG suppressed Ifnb1 expression in the absence of RANKL, it inhibited the decrease in Ifnb1 expression induced by RANKL (Figure 3B), suggesting that this may contribute, in part, to the reduction of c-Fos protein levels by WELG. Taken together, these results suggest that WELG inhibits osteoclast differentiation by suppressing both upregulation of the positive transcription factors and downregulation of the negative transcription factors of osteoclastogenesis.

NF-κB and MAPK (c-Jun N-terminal protein kinase (JNK) and p38 subfamilies) pathways are regarded as the main early signaling events activated during osteoclast differentiation through the involvement of c-Fos and NFATc1 induction [9,10,11]. To further elucidate the inhibitory mechanisms of WELG on osteoclastogenesis, we examined the effects of WELG on RANKL-stimulated MAPKs and NF-κB pathways in BMMs. As shown in Figure 3C, treatment of BMMs with WELG blunted RANKL-induced phosphorylation of JNK and p38 MAPKs. WELG also inhibited RANKL-induced activation of the classical NF-κB pathway, as determined by IκBα phosphorylation and degradation. Thus, these results suggest that the inhibition of WELG on RANKL-induced early signaling events including MAPK and NF-κB pathways may account for its modulatory effects on the expression of the positive and negative osteoclastogenic transcription factors.

### 2.3. Protective Effects of WELG on OVX-Induced Bone Loss and Fat Accumulatioin

**Figure 4:**
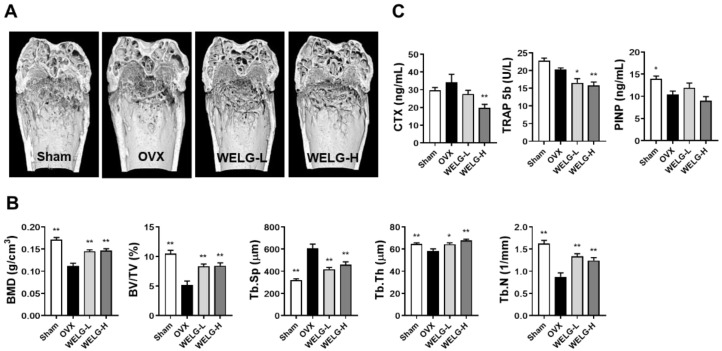
Preventive effects of WELG administration on OVX-induced bone loss in vivo. Mice were orally administered distilled water, WELG-L (100 mg/kg; Low), or WELG-H (300 mg/kg; High) for 6 weeks beginning 1 week after OVX or sham surgery. (**A**) Three-dimensional reconstruction images of the distal femur by micro-computed tomography. (**B**) Morphometric analyses of metaphyseal trabecular bone microstructure. (**C**) Serum levels of CTX, TRAP 5b and PINP. * *p* < 0.05, ** *p* < 0.01 versus OVX group.

In postmenopausal osteoporosis, due to estrogen deficiency, increasing osteoclast formation and activation leads to bone loss [4]. Having confirmed that WELG inhibited RANKL-stimulated osteoclast formation, we investigated the effects of WELG on OVX-induced osteoporosis in vivo. Mice were orally administered WELG for 6 weeks beginning at 1 week after OVX surgery. Three-dimensional images of the distal femur by micro-computed tomography showed that metaphyseal trabecular bone loss was strongly induced in OVX mice compared to that in sham mice. In contrast, trabecular bone loss was recovered by oral administration of WELG at low and high doses (Figure 4A). Quantitative analysis confirmed that trabecular bone mineral density (BMD), bone volume/tissue volume (BV/TV), trabecular thickness (Tb.Th), and trabecular number (Tb.N) in the OVX group were highly decreased compared to those in the sham group. In contrast, trabecular separation (Tb.Sp) in the OVX group was significantly higher than that in the sham group. WELG administration significantly reversed OVX-induced changes in the trabecular bone structural parameters BMD, BV/TV, Tb.N, Tb.Th, and Tb.Sp (Figure 4B). To investigate the mechanisms underlying the inhibitory effects of WELG on bone loss, we measured serum concentrations of C-terminal cross-linked telopeptides of type I collagen (CTX, a bone resorption marker), TRAP 5b as a marker of osteoclast number, and procollagen type I N-terminal propeptide (PINP, a bone resorption marker). Serum CTX levels tended to decrease in the WELG groups compared to those in the OVX group. Similarly, TRAP 5b levels were also reduced in the WELG groups. In contrast, WELG administration did not affect PINP levels decreased by OVX (Figure 4C). Thus, it is likely that the anti-osteoclastic action of WELG might mainly contribute to its protective effect on OVX-induced bone loss.

**Figure 5:**
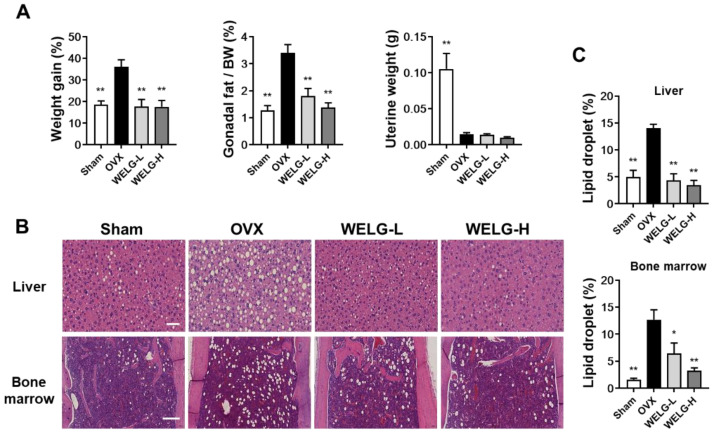
Inhibitory effect of WELG administration on OVX-induced fat accumulation. Mice were orally administered distilled water, WELG-L (100 mg/kg; Low), or WELG-H (300 mg/kg; High) for 6 weeks beginning 1 week after OVX or sham surgery. (**A**) Measurement of body weight changes for 7 weeks, gonadal fat weight/body weight (BW), and uterine weight. (**B**) Representative images of hematoxylin- and eosin-stained liver (scale bar, 50 μm) and bone marrow (scale bar, 200 μm) sections. (**C**) Quantification of lipid droplets in the liver and bone marrow sections. * *p* < 0.05, ** *p* < 0.01 versus OVX group.

Menopause triggers various physiological alterations due to the low transport ratio of estrogen to cholesterol [28,29]. Therefore, the levels of cholesterol in the blood, visceral fat, and body weight increase owing to the strong increase in fat tissues [28,29]. OVX mice showed an intense increase in body weight and gonadal fat weight, which was inhibited by WELG administration (Figure 5A). Osteoporosis is usually associated with increased bone marrow adipose tissue. During osteoporosis development, bone marrow mesenchymal stem cells show a high ability to differentiate into adipocytes rather than osteoblasts, which results in a decrease in bone formation and an increase in marrow fat deposition [30]. It has been shown that increased bone marrow fat inhibits bone healing and regeneration [31]. In this study, OVX significantly increased bone marrow fat accumulation, which was markedly inhibited by WELG administration (Figure 5B,C). Thus, our results suggest that WELG may have beneficial effects on bone by inhibiting both bone loss and bone marrow fat accumulation. In addition to bone marrow fat, WELG also inhibited OVX-induced fat accumulation in the liver (Figure 5B,C).

In the OVX mice model, the weight of the uterus considerably decreases because removal of the ovary led to a decrease in the level of estrogen. In OVX mice, uterine weight significantly decreased compared with sham mice, which was not affected by WELG administration (Figure 5A). These results suggest that the beneficial effects WELG in OVX mice are independent of its potential estrogenic activity.

Our findings demonstrated that WELG has beneficial effects against bone loss, obesity, and ectopic fat deposition induced by OVX. These finding suggest that WELG can be a good candidate for simultaneously managing osteoporosis, obesity, and fat distribution in postmenopausal women.

### 2.4. Phytochemical Profiles of WELG

**Figure 6:**
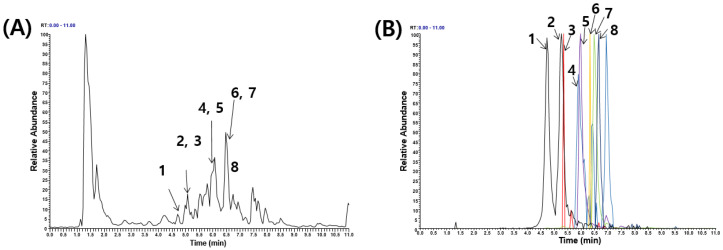
UHPLC–MS/MS analysis of WELG. (**A**) Base peak chromatogram in negative ion mode. (**B**) Extracted ion chromatogram of identified phytochemicals. 1, 5-O-caffeoylquinic acid; 2, chlorogenic acid; 3, feruloylquinic acid; 4, apigenin-6-glucoside-8-xyloside; 5, isoorientin; 6, orientin; 7, p-coumaric acid; 8, isovitexin.

To determine the mechanism of the biological activity and phytochemical constituents of WELG, we investigated its phytochemical profile. The chemical compounds in WELG were detected by ultrahigh-performance liquid chromatography–tandem mass spectrometry (UHPLC–MS/MS) and identified through its MS and MS/MS spectra, comparing with the previously reported MS spectra in a reference library [32,33]. Ultrahigh-performance liquid chromatography–tandem mass spectrometry (UHPLC–MS/MS) analysis of WELG identified four phenolics (5-O-caffeoylquinic acid, feruloylquinic acid, p-coumaric acid, and chlorogenic acid) and four flavonoids (apigenin-6-glucoside-8-xyloside, isoorientin, orientin, and isovitexin), as shown in Table 1. The ion chromatograms for the composition analysis of WELG are shown in Figure 6.

Among these, four phytochemicals have been reported to exhibit potentially beneficial effects on bone. The two flavonoid glycosides, orientin (luteolin 8-C-glucoside) and isovitexin (apigenin-6-C-glucoside), have been shown to stimulate osteoblast differentiation in vitro [34,35]. The two phenolic compounds, p-coumaric acid and chlorogenic acid, have been reported to display a dual activity by inhibiting osteoclast differentiation and stimulating osteoblast differentiation in vitro, as well as to protecting against OVX-induced osteoporosis in vivo [36,37,38,39]. Therefore, it is likely that these active constituents of WELG cooperatively contribute to its anti-osteoclastic and anti-osteoporotic effects.

## 3. Materials and Methods

### 3.1. Materials

Alpha-modified minimum essential medium Eagle (α-MEM) was purchased from Hyclone (Logan, UT, USA). Fetal bovine and calf serums were purchased from Thermo Fisher Scientific (Waltham, MA, USA). AS-MX phosphate, fast red violet LB salt, naphthol, and VitD3 were obtained from Sigma-Aldrich (St. Louis, MO, USA). Lyophilized WELG was obtained from the National Development Institute of Korean Medicine (Gyeongsan, Korea). Briefly, dried leaves of *L. gracile* were extracted by heat reflux extraction with distilled water for 3 h, filtered, and lyophilized. The WELG powder was dissolved in distilled water prior to use.

### 3.2. Isolation and Generation of BMMs

Moue bone marrow was flushed from the femurs and tibias of C57BL/6J mice, and their red blood cells were lysed using ammonium-chloride-potassium buffer. These bone marrow cells were washed with PBS, passed through cell strainers, and then incubated in a complete medium, α-MEM supplemented with 10% FBS and 1% penicillin/streptomycin, supplemented with M-CSF (20 ng/mL) in tissue culture plate. After 24 h, nonadherent cells were cultured in a complete medium supplemented with MCSF (60 ng/mL) in non-coated plates. After five days, the adherent BMMs were dissociated, collected, and used for the osteoclast differentiation assay.

### 3.3. Cell Cytotoxicity Assay

BMMs were cultured for 1 day in the presence of M-CSF (60 ng/mL) with or without WELG (33.3, 100, and 200 μg/mL). Cell cytotoxicity was measured using a CCK-8 reagent (Dojindo Molecular Technologies Inc., Rockville, MD, USA), and absorbance was measured at 450 nm using a microplate reader (Molecular Devices, San Jose, CA, USA).

### 3.4. Osteoclast Differentiation Assay

To examine the effect of WELG on osteoclast differentiation in a BMM–osteocyte co-culture system, MLO-Y4 cells (Kerafast, Boston, MA, USA) cultured for 12 h were co-cultured with BMMs for 5 days with or without WELG (33.3, 100, and 200 μg/mL) in the presence of VitD3 (10 nM). To assess a direct inhibitory effect of WELG on osteoclast differentiation, BMMs were cultured with or without WELG for 4 days in the presence of M-CSF (60 ng/mL) and RANKL (50 ng/mL).

### 3.5. TRAP Staining

Cells were fixed with 10% formalin, permeabilized with 0.1% Triton X-100 in PBS, and then stained with TRAP staining buffer (50 mM sodium tartrate, 0.12 M sodium acetate, naphthol AS-MX phosphate, and fast red violet LB salt, pH 5.2). TRAP-stained cells were washed with distilled water and photographed.

### 3.6. Western Blot Assay

BMMs were harvested and lysed using lysis buffer (iNtRON Biotechnology, Seongnam, Korea). The supernatants were collected as total cell lysates. Protein concentration was quantified using a BCA protein assay kit (Thermo Fisher Scientific, Waltham, MA, USA). Proteins were separated by SDS-PAGE and transferred onto polyvinylidene difluoride (PVDF) membranes. After blocking with 5% skim milk, membranes were incubated with specific primary antibodies (1:1000 dilution) at 4 °C. The primary antibodies are as follows: Phospho-JNK, JNK, phospho-p38, p38, phospho-IκBα, IκBα, and β-actin obtained from Cell Signaling Technology (Danvers, MA, USA); NFATc1 and c-Fos obtained from Santa Cruz Biotechnology, Inc. (Dallas, TX, USA). The membranes were then detected using the corresponding HRP-conjugated secondary antibodies (1:5000 dilution) at room temperature. Finally, chemiluminescent signals were analyzed using a ChemiDoc Imaging System (Bio-Rad, Hercules, CA, USA).

### 3.7. Quantitative Real-Time Polymerase Chain Reaction

Total RNA was isolated using an RNA-spinTM Total RNA Extraction Kit (iNtRON Biotechnology, Seongnam, Korea). cDNA was synthesized from total RNA using a High-Capacity cDNA Reverse Transcription Kit (Thermo Fisher Scientific). The generated cDNA was amplified with TaqMan Universal Master Mix II (Applied Biosystems, Foster City, CA, USA) and TaqMan probes for the target genes Tnfsf11 (Mm00441908_m1), Tnfrsf11b (Mm0043542_m1), c-Fos (Mm00487425_m1), Nfatc1 (Mm00479445_m1), Prdm1 (Mm00476128_m1), Ifnb1 (Mm00439552_s1), Irf8 (Mm00492567_m1), MafB (Mm00627481_s1), Ctsk (Mm00484036_m1), Itgb3 (Mm00443980_m1), Mmp9 (Mm00442991_m1), and 18S rRNA (Hs99999901_s1) using an ABI 7500 Real-Time PCR Instrument (Applied Biosystems).

### 3.8. Animal Study

Seven-week-old C57BL/6J female specific-pathogen-free mice were housed with free access to a standard rodent chow diet and water. Mice were anesthetized with zoletil and rompun, subjected to sham or OVX surgery, and then divided into four groups of six mice each (n = 6/group): sham, OVX, and OVX with low-dose WELG (WELG-L, 100 mg/kg/day) and high-dose WELG (WELG-H, 300 mg/kg/day). One week after OVX surgery, mice were fed a purified low-fat diet (D12450B, Research Diet, New Brunswick, NJ, USA) and orally administered either distilled water, WELG-L, or WELG-H for six weeks. Serum was obtained after sacrifice, and serum levels of CTX, TRAP 5b and PINP were measured using specific ELISA kits (Immunodiagnostic Systems Ltd., London, UK).

For femoral trabecular bone analysis, the distal end of the right femur was scanned using the SkyScan 1276 micro-computed tomography system (Bruker, Kontich, Belgium). The scanned images were reconstructed and analyzed using SkyScan software (Bruker, Kontich, Belgium).

For histological staining, the liver and femur were fixed, dehydrated, embedded into a paraffin block, sectioned into a 5 μm thick slices, and stained with hematoxylin and eosin. The femur was decalcified with RDO Gold (RDO, Aurora, IL, USA) for 1 week prior to dehydration. The stained tissue sections were photographed, and lipid droplet areas were measured using the Image J software.

### 3.9. UHPLC–MS/MS Analysis for Phytochemical Profiles

The WELG was analyzed using a Thermo Dionex UltiMate 3000 HPLC system equipped with a Thermo Q-Exactive mass spectrometer (Thermo Fisher Scientific). An Acquity BEH C18 column (1.7 µm, 100 mm × 2.1 mm) was used for chromatographic separation. The operating protocol was based on a previous study with minor modifications [40,41]. Upon injection of 3-μL samples, they were chromatographically separated using a binary gradient consisting of 0.1% formic acid in water and acetonitrile. The reference standards, orientin, isovitexin, isoorientin, 5-O-caffeoylquinic acid, chlorogenic acid, and p-coumaric acid were obtained from Targetmol (Wellesley Hills, MA, USA).

### 3.10. Statistical Analysis

Data are presented as mean ± standard deviation (SD) for in vitro experiments and as mean ± standard error of the mean (SEM) in animal experiments. The values were assessed by one-way analysis of variance (ANOVA) followed by Dunnett’s test or two-way ANOVA followed by Sidak’s test, and values of *p* < 0.05 were regarded as statistically significant.

## 4. Conclusions

In summary, this study demonstrated that WELG can inhibit osteoclast differentiation and have a protective effect on OVX-induced bone loss. WELG directly inhibited the differentiation of osteoclast precursors into osteoclasts by suppressing RANKL-induced activation of MAPKs and NF-κB, upregulation of positive regulators, and downregulation of negative regulators of osteoclastogenesis. In an OVX-induced postmenopausal osteoporosis mouse model, WELG administration prevented bone loss, weight gain, and ectopic fat accumulation without affecting uterine atrophy. Furthermore, we identified active constituents which have bone-protective effects in WELG. Taken together, our findings suggest that WELG might be a potential candidate for the simultaneous management of osteoporosis and obesity in postmenopausal women.

## Figures and Tables

**Table 1:** The phytochemicals present in WELG as analyzed by UHPLC–MS/MS.

No	t_R_ (min)	[M − H]^−^ (*m*/*z*)		Elemental Composition	Error (ppm)	MS/MS Fragments (*m*/*z*)	Identification
		Estimated	Calculated				
1	4.70	353.0878	353.0877	C_16_H_18_O_9_	2.808	191.0551, 179.0338, 135.0437	5-O-Caffeoylquinic acid *
2	5.25	353.0878	353.0876	C_16_H_18_O_9_	2.525	191.0550, 179.0338, 173.0443, 135.0436	Chlorogenic acid *
3	5.34	367.1050	367.1032	C_17_H_20_O_9_	2.292	193.0495, 173.0444, 149.0593, 134.0358	Feruloylquinic acid
4	5.93	563.1392	563.1399	C_26_H_28_O_14_	0.654	563.1392, 503.1185, 473.1083, 443.0967, 383.0767, 353.0669	Apigenin-6-glucoside-8-xyloside
5	5.97	447.0933	447.0930	C_21_H_20_O_11_	2.657	357.0612, 327.0507	Isoorientin *
6	6.33	447.0933	447.0930	C_21_H_20_O_11_	1.474	357.0613, 327.0508, 285.0398	Orientin *
7	6.47	163.0546	163.0387	C_9_H_8_O_3_	1.660	163.0388, 120.0520, 119.0486	p-Coumaric acid *
8	6.65	431.0989	431.0980	C_21_H_20_O_10_	1.686	341.0662, 311.0558	Isovitexin *

* compared with the retention time and MS spectral data of authentic standards.

## Data Availability

All data of this study are provided within this study.
